# Print Media Response to SARS in New Zealand

**DOI:** 10.3201/eid1008.031096

**Published:** 2004-08

**Authors:** Nick Wilson, George Thomson, Osman Mansoor

**Affiliations:** * Otago University, Wellington, New Zealand;; † Public Health Consulting Ltd, Wellington, New Zealand

**Keywords:** SARS virus, newspapers, health promotion, risk, risk reduction behaviour, communication, travel, dispatch

## Abstract

To examine the media response to severe acute respiratory syndrome, we reviewed New Zealand's major newspaper (261 articles for 3 months). While important accurate health messages were frequently included, some were missed (e.g., hand washing in only 2% of articles). No incorrect information was identified, and health spokespersons were accurately quoted.

Severe acute respiratory syndrome (SARS) is a new viral disease in humans that emerged in southern China in November 2002 (1). The World Health Organization (WHO) issued a global alert about SARS on March 12, 2003, and an unprecedented public health response was subsequently mounted. An important part of that response was probably the intense global media coverage given to this disease. To derive lessons for addressing future threats to public health, we examined the media response in New Zealand's major daily newspaper.

## The Study

We searched the Internet-based electronic archive of the New Zealand Herald for a 3-month period beginning with WHO's first global alert (March 13–June 11, 2003). We chose this paper because it has the largest circulation of a daily paper in the country (i.e., 530,000 readers out of a national population of 4 million), and its reporting is likely to represent that of other mainstream media. The advanced search capacity at the newspaper's Web site (2) was used with the key search term being "SARS" (combined with the other terms detailed in Tables 1 and 2 [3–5]; ). The search was confined to the news section of the archive because stories on SARS in the business and sports sections rarely provided information on health aspects.

**Table 1:** Information on the clinical features of SARS in the New Zealand Herald^a^

Clinical feature	No. (%) of articles (N = 261)
Symptoms detailed on the Ministry of Health's SARS Web site	
Cough or fever	67 (26)
Cough	55 (21)
Fever^b^	54 (21)
"Shortness of breath"	15 (6)
"Trouble breathing" or "difficulty breathing"	5 (2)
"Body aches" or "muscle pain" (myalgia^b^)	3 (1)
"Diarrhoea"^b^ or "discomfort"	2 (1)
Additional symptoms of SARS from the literature (3–5)	
Chills^b^	12 (5)
Headache	5 (2)
Other^c^	3 (1)
Other words relating to clinical features	
"Pneumonia" or "flu"	103 (39)
Pneumonia	67 (26)
Flu	53 (20)
Flu-like	38 (15)
Influenza	17 (7)
"High fever"	34 (13)
Temperature	23 (9)
Temperature of 38°C	9 (3)
"High temperature"	6 (2)
"Respiratory symptoms"	3 (1)

^a^SARS, severe acute respiratory syndrome. Quotation marks refer to actual phrases in newspaper articles. ^b^These were the symptoms considered to predict SARS most strongly in the early stages of illness, according to Rainer et al. (4). ^c^Other signs and symptoms included loss of appetite, malaise, rigor, vomiting, sore throat, dizziness, sputum, night sweat, coryza, abdominal pain, neck pain, nausea, arthralgia ("joint pain"), chest pain, rhinorrhea ("runny nose").

**Table 2:** Information on SARS transmission and control measures reported in the New Zealand Herald^a^

Information on SARS	No. (%) of articles (N = 261)
SARS transmission	
Transmission by "droplets" or "sneezing" or "coughing"	16 (6)
"Close contact," "direct contact," or "physical contact" with an infected person as a risk factor for transmission	16 (6)
"Close contact" or "contacts" the definition used for outbreak control purposes	13 (5)
Possible transmission through a contaminated "surface" or "object" or lift "button" or door "handle"	13 (5)
"Person-to-person" transmission	7 (3)
Possible risk posed by bodily "secretions" (or "faecal" contamination, "faeces" or "stool")	7 (3)
Possibility of "airborne" transmission	5 (2)
"Casual contact" not being a risk factor for transmission	2 (1)
No evidence for "airborne" transmission (or unlikely)	2 (1)
Touching one's "eyes," or "nose," or "mouth" with potentially contaminated hands as a risk factor	1 (0.4)
SARS control or personal protection	
"Quarantine"	85 (33)
"Isolation"	62 (24)
"Mask"	60 (23)
"Hand washing" for prevention	4 (2)
Advice to seek medical attention if relevant symptoms are present	4 (2)
Lack of health insurance coverage for travellers to affected areas	2 (1)
Groups at increased risk of infection and or death	
Health workers (including nurses and doctors)	24 (9)
"Elderly" (and other terms for older persons)	7 (3)
Persons with diabetes or other chronic conditions	3 (1)

^a^SARS, severe acute respiratory syndrome. Quotation marks refer to actual phrases used in newspaper articles.

We compared information in the articles on SARS with that in the Medline-indexed literature (to July 2003). Information attributable to health officials in New Zealand was compared to the information on the Ministry of Health's Web site and its media releases (n = 19) (6). For comparison purposes, we obtained from WHO the weekly numbers of new cases of SARS from four areas that had ongoing SARS transmission in the Western Pacific Region; China was excluded because of its irregular pattern of reporting.

SARS dominated the health-related news in this newspaper during the study period, with 261 news articles (i.e., 3.3 articles per issue). The rate of articles mentioning SARS (87 per month) was greater than that for smoking and tobacco (59 per month), cancer (43 articles), diabetes (12 articles), heart disease (10 articles), and asthma (6 articles). The number of articles mentioning SARS rose and fell, more or less in line with disease activity (Figure). Of the 261 articles, 48% had a headline with the word SARS.

**Figure:**
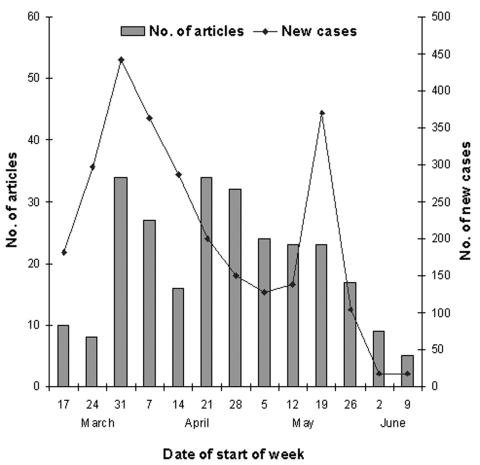
Articles on severe acute respiratory syndrome (SARS) in the New Zealand Herald and new cases of SARS (Singapore, Hong Kong, Vietnam, Taiwan).

In the 261 articles, no technically incorrect information about the clinical or epidemiologic features of SARS appeared in print. Also, the views or comments attributed to Ministry of Health spokespersons were consistent with messages promoted by the ministry in its media releases and on its SARS Web site (31 articles). The impression conveyed was that the spokespersons were credible, and their reported statements imparted information and reassurance, and sometimes put the risk for SARS into a broader risk perspective. Some statements by officials promoted the theme of civic responsibility by stating that persons who ignored official travel advisories were placing others at risk.

The symptoms of cough or fever were mentioned in 26% of articles, and the terms "pneumonia" or "flu" were also commonly used (39%) (Table 1). The word "flu" was used in the articles far more frequently than "influenza." More detailed information on symptoms was rare; 3% of articles mentioned the specific temperature of 38°C (the figure used in official information).

All the countries with in-country transmission of SARS were mentioned, with particular emphasis on China (65% of articles). Travel advice from official agencies was from WHO (12% of articles), the Ministry of Health (8%), and other government agencies (4%). However, few articles included the specific ministry advice that recent travelers from SARS-affected countries avoid nonessential medical visits but seek medical attention if relevant symptoms developed (Table 2).

Articles frequently described public health responses involving quarantine or isolation (Table 2). Masks were frequently mentioned (or shown in photographs), and information on disease transmission was sometimes provided. This information appeared to be accurate, although occasionally unconfirmed means of transmitting SARS (e.g., airborne spread, as opposed to spread by droplets from coughing, and spread through cockroaches and rodent feces) were mentioned. Eight articles (3%) provided a Web site address for SARS information. The Ministry's SARS Web site and three relevant 0800 telephone helplines were infrequently mentioned (n = 4 [2%] and n = 8 [3%], respectively).

In retrospect, some comments reported were overly pessimistic. For example, an economist was reported as saying that the disease "was on its way to New Zealand, and once here it was unlikely to go away quickly." International health officials were also quoted as saying that SARS "is probably here to stay" and "is now probably entrenched in the population [in China]."

Particular terms were used that could be considered alarming (e.g., "outbreak" in 38% of articles, "deadly" in 32%). Similarly, at least one of the following terms was used in 15% of headlines: kill, killer, deadly, panic, and death (n = 38). Some examples of headline phrasing included the following: "doctor dies of killer virus"; "nature's terrorism strangles Hong Kong"; "SARS deaths leap"; "panicking crowds flee"; "creeping panic over epidemic"; and "SARS virus… mutating rapidly."

## Discussion

This analysis is limited by its focus on only one New Zealand newspaper (albeit the one with the largest daily circulation). It also lacks the broader context that could have been obtained from interviews with key personnel. Nevertheless, it provides some insight into the media response to an emerging public health threat.

SARS clearly dominated the health news during this study period, although total coverage was possibly limited by competition from another major event, the war in Iraq. This media interest might be due to a number of newsworthy features concerning SARS, namely, its new disease status, exotic aspects (e.g., possibly arising from wild animals), relative infectiousness, uncertain transmission modes, high case-fatality rate, and limited treatment options.

Information on disease symptoms was frequently provided but often with little accompanying detail. This finding highlights the importance of health authorities' keeping key messages short and using well-published Web sites for providing more detail. The media appear to be much more likely to use some words than others (e.g., "flu" versus "influenza" and "outbreak" versus "pandemic"). This finding suggests the need for health authorities to use simple language and to use it consistently.

Although some prominence was given to describing disease control interventions (e.g., quarantine and isolation), relatively few articles provided information on basic personal preventive measures such as hand washing. Little coverage was given to how to access Web sites or telephone helplines, perhaps because newspapers only partly see themselves as a "public good information service" and may expect health authorities to pay to advertise such details. This finding suggests that if critical health messages are not picked up by the media in a crisis, then paid advertising could be a backup option (especially to list and explain access to key Web sites). Indeed, health budgets could ideally take into account such contingencies.

## Conclusions

This analysis showed that official health spokespersons were accurately quoted and that no technically incorrect information on the clinical or epidemiologic features of SARS was published. Such a response is reassuring and highlights the potential value of the health sector's use of the media to inform the public.

This newspaper sometimes used headlines and particular words (e.g., "deadly") that could be considered alarming. Nevertheless, some articles quoting health officials and others did provide reassuring information and messages.

Media analyses could be extended in a number of ways to provide a broader and deeper understanding of the response to SARS. A range of newspapers could be studied, along with key interviews with health reporters and health sector spokespersons. Such work could be justified, given the importance of risk communication in dealing with the possible reemergence of SARS (7) and the importance of information for the public and the media on the control of this disease (8). These issues are also relevant in handling the threat of pandemic influenza (9) or diseases associated with bioweapons that could spread globally (10).

In summary, this particular major daily newspaper provided generally useful and accurate information to the public on SARS. This finding highlights the potential value of the media for communication about public health issues and pandemic threats.

We thank the New Zealand Ministry of Health for supporting the initial phase of this work, which was undertaken when the first author was working on SARS control for the Minstry. The findings are those of the authors and do not reflect the views of the Ministry of Health.

## References

[1] Pearson H, Clarke T, Abbott A, Knight J, Cyranoski D. SARS: What have we learned? Nature. 2003;424:121–6. 10.1038/424121a 12853923 PMC7095391

[2] New Zealand Herald. New Zealand Herald article search (advanced). [accessed 2003 Dec 14]. Available from: http://www.nzherald.co.nz/storyquery.cfm

[3] Donnelly CA, Ghani AC, Leung GM, Hedley AJ, Fraser C, Riley S, Epidemiological determinants of spread of causal agent of severe acute respiratory syndrome in Hong Kong. Lancet. 2003;361:1761–6. 10.1016/S0140-6736(03)13410-1 12781533 PMC7112380

[4] Rainer TH, Cameron PA, Smit D, Ong KL, Hung AN, Nin DC, Evaluation of WHO criteria for identifying patients with severe acute respiratory syndrome out of hospital: prospective observational study. BMJ. 2003;326:1354–8. 10.1136/bmj.326.7403.1354 12816820 PMC162123

[5] Booth CM, Matukas LM, Tomlinson GA, Rachlis AR, Rose DB, Dwosh HA, Clinical features and short-term outcomes of 144 patients with SARS in the Greater Toronto area. JAMA. 2003;289:2801–9. 10.1001/jama.289.21.JOC30885 12734147

[6] New Zealand Ministry of Health. SARS information archive. [Accessed 2003 Jul 3]. Available from: http://www.moh.govt.nz/moh.nsf/f872666357c511eb4c25666d000c8888/798fe182730a9f37cc256d8000758927?OpenDocument

[7] Pirisi A. Getting ready for SARS. Lancet. 2003;362:1632–3. 10.1016/S0140-6736(03)14827-1 14631962 PMC7135301

[8] Lau JTF, Yang X, Tsui H, Kim JH. Monitoring community responses to the SARS epidemic in Hong Kong: from day 10 to day 62. J Epidemiol Community Health. 2003;57:864–70. 10.1136/jech.57.11.864 14600111 PMC1732318

[9] Ministry of Health. Influenza pandemic action plan. Wellington: Ministry of Health, 2002. [Accessed 2003 Jun 19]. Available from: http://www.moh.govt.nz/moh.nsf/ea6005dc347e7bd44c2566a40079ae6f/5f5694e4a5736dd2cc256c55000788a3/$FILE/InfluenzaPandemicActionPlan.pdf

[10] Wilson N, Lush D. Bioterrorism in the Northern Hemisphere and potential impact on New Zealand. N Z Med J. 2002;115:247–51. 12117179

